# Important roles of multiple Sp1 binding sites and epigenetic modifications in the regulation of the methionine sulfoxide reductase B1 (MsrB1) promoter

**DOI:** 10.1186/1471-2199-8-39

**Published:** 2007-05-22

**Authors:** Antonella De Luca, Paolo Sacchetta, Marzia Nieddu, Carmine Di Ilio, Bartolo Favaloro

**Affiliations:** 1 From the Department of Biomedical Sciences, University of Chieti "G. D'Annunzio" School of Medicine, and Unit of Gene Regulation, Center of Excellence on Aging, "G. D'Annunzio" University Foundation, Chieti, Italy

## Abstract

**Background:**

Methionine sulfoxide reductases (Msrs) are enzymes that catalyze the reduction of oxidized methionine residues. Most organisms that were genetically modified to lack the *MsrA *gene have shown shortening of their life span. Methionine sulfoxide reductases B (MsrB) proteins codified by three separate genes, named *MsrB1*, *MsrB2*, and *MsrB3*, are included in the Msrs system. To date, the mechanisms responsible for the transcriptional regulation of *MsrB *genes have not been reported. The aim of this study was to investigate the regulation of MsrB1 selenoprotein levels through transcriptional regulation of the *MsrB1 *gene in MDA-MB231 and MCF-7 breast carcinoma cell lines.

**Results:**

A *MsrB1 *gene promoter is located 169 base pairs upstream from the transcription start site. It contains three Sp1 binding sites which are sufficient for maximal promoter activity in transient transfection experiments.

High levels of MsrB1 transcript, protein and promoter activity were detected in low metastatic MCF7 human breast cancer cells. On the contrary, very low levels of both MsrB1 transcript and promoter activity were detected in the highly metastatic counterpart MDA-MB231 cells.

A pivotal role for Sp1 in the constitutive expression of the *MsrB1 *gene was demonstrated through transient expression of mutant *MsrB1 *promoter-reporter gene constructs and chromatin immunoprecipitation experiments.

Since Sp1 is ubiquitously expressed, these sites, while necessary, are not sufficient to explain the patterns of gene expression of *MsrB1 *in various human breast cancer cells. MDA-MB231 cells can be induced to express MsrB1 by treatment with 5-Aza-2'-deoxycytidine, a demethylating agent. Therefore, the *MsrB1 *promoter is controlled by epigenetic modifications.

**Conclusion:**

The results of this study provide the first insights into the transcriptional regulation of the human *MsrB1 *gene, including the discovery that the Sp1 transcription factor may play a central role in its expression. We also demonstrated that the *MsrB1 *promoter activity appears to be controlled by epigenetic modifications such as methylation.

## Background

The methionine sulfoxide reductases enzymes (Msrs) family contains proteins that can reduce both free and protein-linked oxidized methionine residues. To date, the two Msr enzymes that reduce the epimeric forms of methionine sulfoxide Met(O) in proteins are referred to as MsrA and MsrB: they catalyze the reduction of methionine-S-sulfoxide (Met-SO) and methionine-R-sulfoxide (Met-RO), respectively [1-3]. Several authors reported that MsrA and MsrB require reduced thioredoxin as the natural reducing system, although DTT can be used *in vitro *[1,3,4]. As reported by Sagher *et al*., thioredoxin is a poor reducing agent for both hMsrB2 and hMsrB3 [5]. However, in the presence of selenocystamine, the thioredoxin reducing system is very effective with MsrB enzymes. A cysteine residue in the N-terminal region of the MsrB1 is important for the thioredoxin-recycling process of the MsrB1 [6]. Recently, a thionein derived from metallothionein and identified in bovine liver was shown to support Msr activity in absence of either thioredoxin or DTT (7).

Oxidation of methionines may affect protein function and was implicated in several processes, such as oxidative stress, accelerated aging, and neurodegenerative diseases [8-12]. MsrA has been studied in detail for several years and is well characterized structurally and functionally. The importance of this enzyme was highlighted by several studies. *Escherichia coli *MsrA mutant seemed to be more sensitive to oxidative damage than the parent strain [13]. Abolishing the MsrA gene in yeast caused enhanced accumulation of methionine oxidation, while shortening their survival rate in response to oxidative stress [14,15]. *Msra*-knockout mice had a shorter life span, were more sensitive to hyperbaric oxygen, and had a neurological defect that resulted in abnormal walking [16]. Overexpression of MsrA nearly doubled the life span in *Drosophila melanogaster *[17]. Moreover, MsrA activity, as well as gene and protein expression, decreased as a function of age [18,19].

Human genome contains one single *MsrA *gene and three separate genes named *MsrB1*, *MsrB2*, and *MsrB3: the latter *codifying for MsrB3A and MsrB3B. These proteins have a specific sub-cellular localization: MsrB1 is located in the cytoplasm and nucleus, MsrB2 and MsrB3B in mitochondria and MsrB3A in the endoplasmic reticulum [20].

MsrB1 is a selenoprotein also known as selenoprotein R or selenoprotein X. Selenocysteine (Sec) plays a crucial role in MsrB1 enzyme activity [6,20,21]. MsrB1 possessed a 100-fold higher activity than its cysteine mutant form. Moreover, substitution of catalytic cysteine with Sec resulted in more than 100-fold increased activities in the non-selenoprotein MsrB2 and MsrB3[22]. Among the MsrB proteins, MsrB1 possessed the highest specific activity and was considered, together with MsrA, the enzyme that contributed significantly to the reduction of methionine sulfoxides in cells [20].

The overproduction of reactive oxygen species (ROS) can result in various deleterious effects. Aerobic organisms have developed a number of cellular defences as a protection against these harmful ROS [23]. The classical cellular defence against ROS includes enzymes such as catalase (CAT), superoxide dismutases (SOD), and glutathione peroxidase (GPX). In recent years, the Msr system, which can repair oxidative damage to proteins, has been added to this important "antioxidant triad". The Msr system is based on enzymes that can reduce both free and protein-linked oxidized methionine residues; thus this system is now considered an important defence mechanism against oxidative damage [1,9,24,25].

Despite the importance of Msr enzymes in the defence system against oxidative damage, little is known about the transcriptional regulation of their genes. Moskovitz *et al*. reported that mRNA and protein levels of MsrB1 were associated with MsrA expression, as well as to selenium supply [26]. However, the mechanism by which MsrA might have a role in *MsrB1 *regulation has not been elucidated.

We recently reported the identification and analysis of the promoter region of the human *MsrA *gene [27]. The MsrA expression appeared to be different in several human cancer cell lines and *MsrA *gene promoter analysis showed a cell-specific transcriptional regulation of this gene [27].

In this study, we investigated the transcriptional regulation of the *MsrB1 *gene in human breast cancer cells. We found different MsrB1 expression levels in cancer cell lines derived from the same tissue. We determined the transcription start site (TSS) and isolated the 5'-flanking region of the *MsrB1 *gene. We showed that multiple Sp1 binding sites were required for promoter activity and the observed difference in *MsrB1 *gene expression could be explained as a consequence of epigenetic modifications.

## Results

### MsrB1 transcript and protein are differentially expressed in breast cancer cell lines

**Figure 1:**
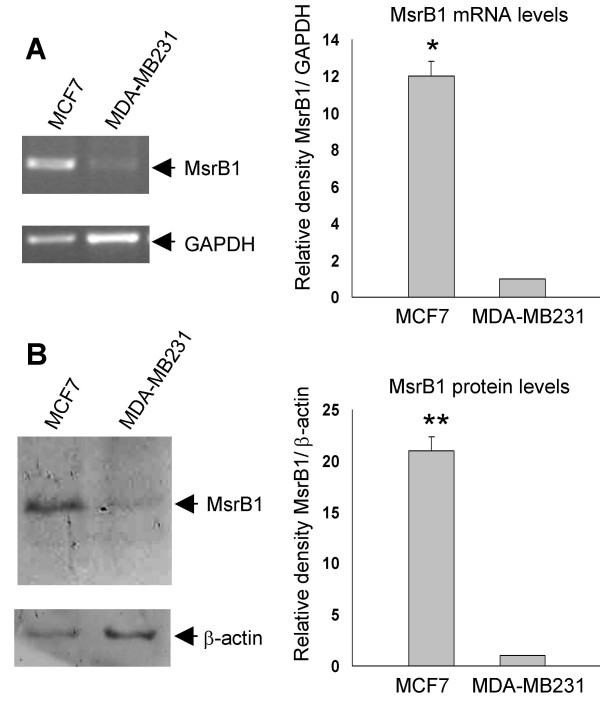
**Analysis of MsrB1 transcript and protein levels in MCF7 and MDA-MB231 breast cancer cell lines**. **(A) **Ethidium bromide-stained gel showing the level of MsrB1 transcript between MCF7 and MDA-MB231 cells. GAPDH was used as the control. The MsrB1 transcript levels in MCF7 and MDA-MB231 cells were analysed using Chemi Doc System (Bio-Rad) and normalized by GAPDH signal intensities of the corresponding lanes. The data are reported as mean ± S.D. of three independent experiments; * P < 0.001. **(B) **Immunoblotting of MCF7 and MDA-MB231 extracts with a mouse monoclonal anti-MsrB1. The MsrB1 protein (13 kDa) levels in MCF7 and MDA-MB231 cells were analysed using Chemi Doc System (Bio-Rad) and normalized by β-actin (42 kDa) signal intensities of the corresponding lanes. Values are reported as mean ± S.D. of at least five different immunoblotting experiments; ** P < 0.005.

In order to evaluate the relative levels of MsrB1 transcript in both MDA-MB231 and MCF7 cells, RT-PCR was conducted using 1 μg of total RNA samples obtained from these cells. As shown in Figure 1, a lower level of MsrB1 transcript was detected in RT-PCR products obtained using total RNA extracted from MDA-MB231 cells compared to those obtained from MCF7 cells. Levels of MsrB1 protein detected by Western blot analysis were consistent with the MsrB1 transcript data (Figure 1). In fact, a lower level of MsrB1 protein was detected in crude extracts obtained from MDA-MB231 cells versus MCF7 cells, suggesting a different *MsrB1 *transcriptional regulation in these human breast cancer cell lines.

### Identification of transcription start site

**Figure 2:**
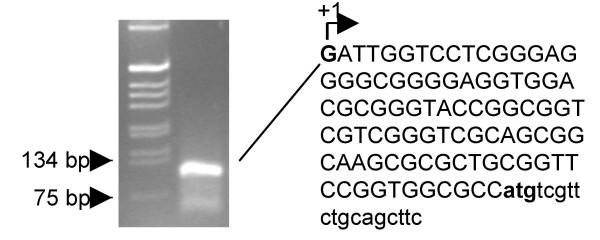
**5' – RACE analysis of the MsrB1 gene**. Agarose gel electrophoresis of size marker and MsrB1 5'-RACE product from purified total RNA using primer RACE -2 (Table 1). The 5' terminal of the sequence of the band from the RACE -2 primer reaction is reported. The upstream sequence from the fragment cloned from the band is in upper case and the arrow indicates the transcription start site (+ 1). The open reading frame codifying for MsrB1 is in lower case and the start codon is in boldface type.

To identify the transcription start site (TSS) of the *MsrB1 *gene, we performed 5' RACE analysis using total RNA isolated from MCF7 cells. The analysis was performed using two reverse oligonucleotides derived from the 5'-UTR region (Table 1) and two adapter primers provided with the kit for the primary and nested PCR. A weak smear on agarose gel was observed in the first PCR with outer primer GeneRacer™ 5' Primer and reverse primer RACE-1, using the total RNA from MCF7 cells. The primary PCR products were used as templates for nested PCR using GeneRacer™ 5' Nested Primer and RACE-2 nested primer. Agarose gel electrophoresis resolved the 5'-RACE-nested reaction product into a single DNA fragment (Figure 2) which was then cloned. The sequencing of six different randomly-selected clones showed the same sequence (Figure 2). The TSS was identified by sequence analysis and mapped 90 bp upstream of the first ATG translation codon.

### MsrB1 promoter activity in transient transfection of different cell types

**Figure 3:**
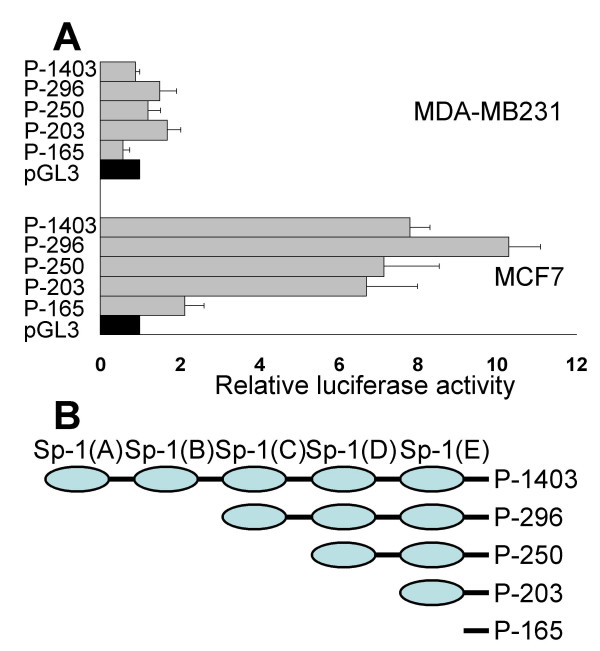
**Analysis of MsrB1 promoter function in breast cancer cell lines**. (**A**) MCF7 and MDA-MB231 cells were transiently transfected with five promoter constructs ranging from nt -1276 (P-1403) to -38 (P-165) relative to transcription start site and extending to nt +127. Promoter fragments were inserted into the pGL3-Basic luciferase vector. Luciferase activity was measured as relative light units of firefly luciferase normalized with respect to protein concentration. Promoter activity is expressed as the magnitude of normalized luciferase activity relative to the pGL3-Basic promoterless negative control vector (pGL3). The results are reported as mean ± S.D. of at least five different experiments, in duplicate for each construct. (**B**) a schematic map of the deletion constructs excluding each Sp1 binding site is reported (Sp-1A, Sp-1B, Sp-1C, Sp-1D and Sp-1E).

On the basis of the TSS position, a 1403 bp fragment of human genomic DNA (-1276/+127) was obtained by PCR. The sequence of the 5'-flanking region of the *MsrB1 *gene was analysed using TFSEARCH software for the presence of transcription binding sites. The *MsrB1 *5'-flanking region revealed no canonical TATA and/or CAAT boxes, but did contain several putative binding sites for Sp1 and showed a high content of CpG islands. To map promoter activity of the 5'-flanking region of the *MsrB1 *gene, various 5' progressive deletions excluding each Sp1 binding site starting from -1276 were prepared. The 1403 bp DNA fragment was subcloned, sequenced and used as template to make PCR products of lengths between -1276/+127 (P-1403) and -38/+127 (P-165). These DNA-fragments were subcloned into the promoterless pGL3 Basic reporter plasmid and the resulting P-1403, P-296, P-250, P-203, and P-165 constructs were analysed by sequencing to ensure fidelity of amplification. The human MCF7 and MDA-MB231 cells were chosen as breast cancer cell models for the analysis of the *MsrB1 *promoter activity. Figure 3 shows the relative luciferase activities of reporter constructs transfected in these cells. Maximal promoter activity was detected, using the P-296 construct, 169 bp (containing the three Sp1 sites) from the TSS. Constructs longer than 296 bp had reduced activity suggesting the presence of possible cis-elements recognized by transcription factors. However, computational analysis (TFSEARCH) of the *MsrB1 *promoter did not show the presence of distinct upstream inhibitory elements. We detected very low levels of MsrB1 mRNA in MDA-MB231 cells and in accordance a very low promoter activity detected in these cells (Figure 1).

### Site-directed mutagenesis of the MsrB1 promoter

**Figure 4:**
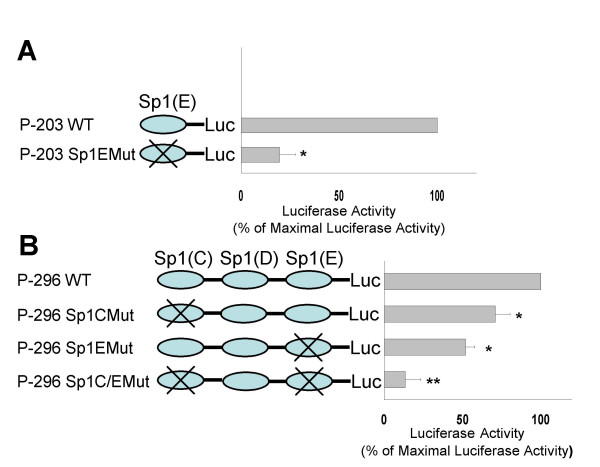
**Effect on the transcriptional activity of site-directed mutagenesis of the putative Sp1-binding site of the MsrB1 promoter**. (**A**) the sequence spanning Sp1- binding site (Sp1E) contained in the construct P-203 WT was subjected to site-directed mutagenesis. The mutated construct was designed P-203 Sp1EMut. Luciferase reporter activity was assessed following transfection into MCF7 cells. Luciferase activity was inhibited by approximately 80% by mutation of Sp1E site. (**B**) the sequences spanning Sp1-binding sites (Sp1C and Sp1E) contained in the P-296 WT construct were subjected to site-directed mutagenesis. The constructs obtained were designated as P-296 Sp1CMut and P-296 Sp1EMut. In addition a double mutant containing both mutations was indicated as P-296 Sp1C/EMut. The mutated constructs were transiently expressed in MCF7 cells for luciferase assays. Luciferase activity was inhibited by approximately 30% by mutation of Sp1C site; 24% by mutation of Sp1E site and by approximately 76% by mutation of both Sp1 sites (Sp1C/Sp1E). The results are expressed as mean ± S.D. of at least five different experiments, in duplicate for each construct. Statistically significant differences compared to the appropriate WT construct are indicated by *P < 0.005 and **P < 0.002.

The contribution of Sp1 transcription factor in *MsrB1 *regulation was assessed by site-directed mutagenesis of their binding sites within the *MsrB1 *promoter-reporter gene constructs (P-203 and P-296). On the basis of the transient transfection experiments reported above, we focused our attention on the Sp1 sites named Sp1E and Sp1C (Figure 3). Reporter constructs containing mutated sequence for Sp1E site, P-203 Sp1EMut, as well as P-203 clone wild-type, were transfected into MCF7 cells. Mutating Sp1E site brought the promoter activity down to basal level, suggesting that this site is important for the promoter activity detected using P-203 construct (Figure 4). Our experiments also revealed that both the Sp1 sites regulated the *MsrB1 *transcription. The mutation of either Sp1E or Sp1C sites produced a decrease in promoter activity (Figure 4). Moreover, transfection experiments using P-296 Sp1C/EMut construct, containing mutations of both Sp1 binding sites, produced a dramatic decrease in the maximal promoter activity (Figure 4).

### Chromatin immunoprecipitation locates Sp1 to the MsrB1 promoter in vivo

**Figure 5:**
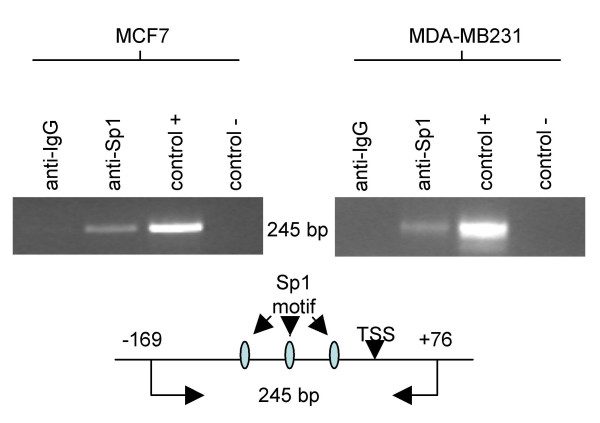
**Chromatin immunoprecipitation assay performed on MCF7, and MDA-MB231 breast cancer cell lines to evaluate in vivo Sp1 binding**. Proteins were cross-linked to the DNA with formaldehyde and Abs directed against RNA-Pol (Ab control) or Sp1 were added to precipitate any protein-DNA complexes. The control + lane was DNA that had been sonicated and pre-cleared with protein G beads. The control-lanes were processed according to the protocol, but did not have any Ab added to the samples. PCR were performed on isolated DNA using primers encompassing MsrB1 promoter region -169 to +76. A schematic map of the amplified DNA fragment (245 bp) containing Sp1 binding motifs and TSS position is illustrated as well.

**Figure 6:**
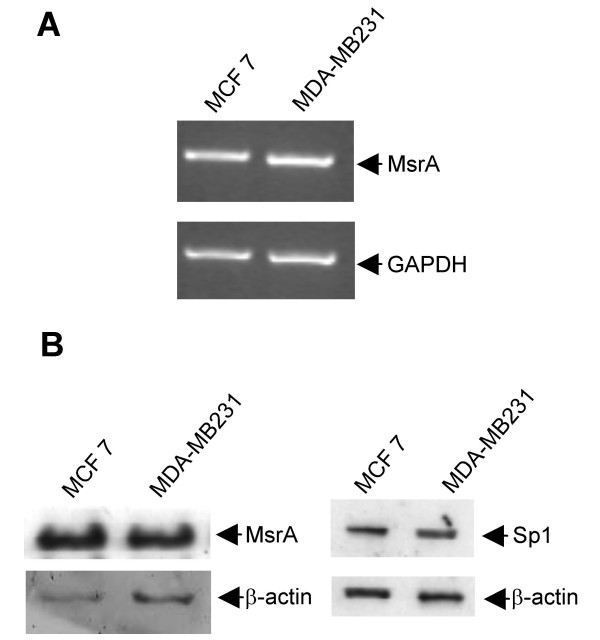
**Expression of MsrA and Sp1 in MCF7 and MDA-MB231 cells**. (**A**) MsrA mRNA levels were evaluated after RT-PCR of mRNA samples obtained from MCF7 and MDA-MB231 cells, as described. (**B**) a representative Western blot showing MsrA (26 kDa) and Sp1 (105 kDa) protein levels.

Chromatin immunoprecipitation (ChIP) assays were performed in MCF7 and MDA-MB231 cells to further elucidate the mechanisms of *MsrB1 *promoter regulation. A promoter *MsrB1*-specific PCR was performed on sheared chromatin, which was immunoprecipitated with an Sp1-specific antibody. As shown in Figure 5, a specific PCR product was obtained using primers encompassing *MsrB1 *promoter region -169 to +76. It is interesting to note that this region contained three Sp1 binding sites (Figure 3). The *MsrB1 *promoter PCR fragment was obtained from both breast cancer cell lines. These results indicated a direct *in vivo *interaction of Sp1 with the *MsrB1 *promoter in both MCF7 and MDA-MB231 cells. Similar Sp1 levels were detected by Western blot analysis in both cell lines (Figure 6).

### MsrA does not seem to be involved in MsrB1 expression in breast cancer cells

Since MsrA could have a role in *MsrB1 *transcription [26], we compared both the MsrA mRNA and protein levels in high-expressing MsrB1 MCF7 cells and low-expressing MDA-MB231 cells. As reported in Figure 6, comparable levels of MsrA transcript were detected in both the breast cancer cells tested. In addition, similar levels of MsrA protein were observed. These results suggested that *MsrB1 *expression was not influenced by the MsrA expression levels.

### Effects of DNA demethylation and histone deacetylase inhibition on MsrB1 expression

**Figure 7:**
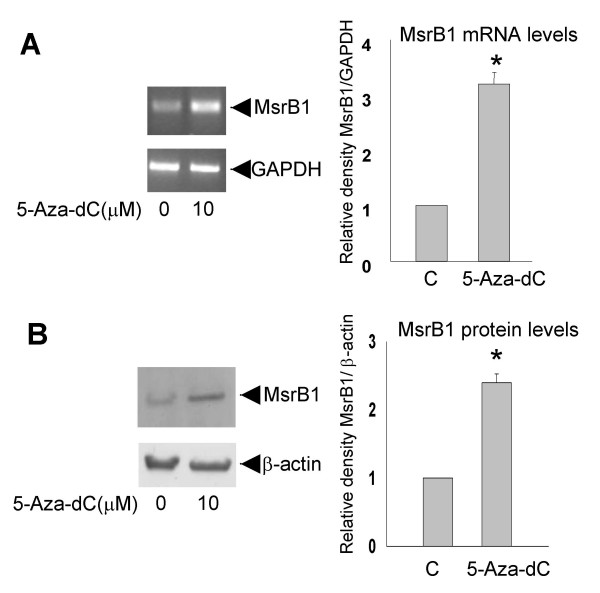
**Induction of MsrB1 by 5-Aza-dC**. (**A**) MDA-MB231 cells were treated with 10 μM 5-Aza-dC as described. MsrB1 expression was detected by RT-PCR. The change in MsrB1 mRNA level is indicated relative to the basal (non treated) level of MsrB1 mRNA. GAPDH mRNA was amplified as a control. (**B**) whole cell extracts were prepared from MDA-MB231 cells treated with 10 μM 5-Aza-dC and subjected to immunoblotting for MsrB1 protein expression. The membrane filters were re-probed with anti-β-actin antibody to verify the equal loading and integrity of samples. Data shown are representative of five independent experiments. The results are reported as mean ± S.D.; * P < 0.01.

**Figure 8:**
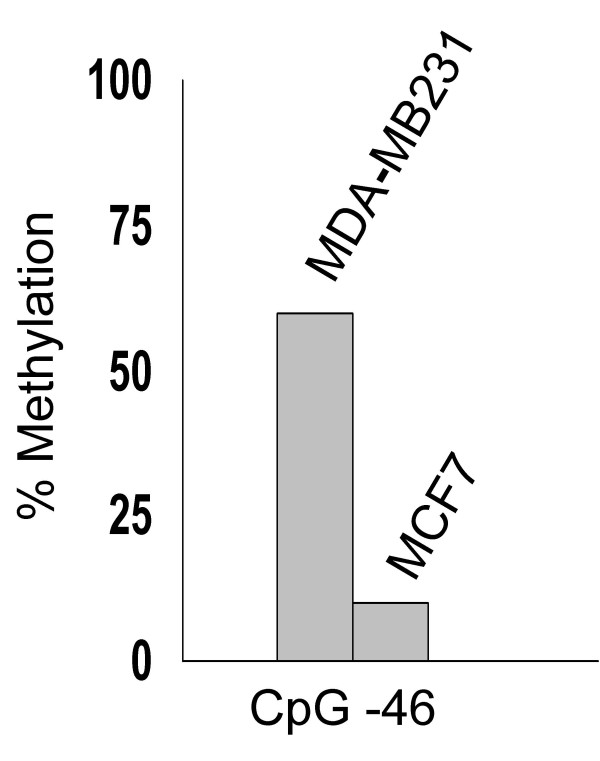
**Analysis of MsrB1 CpG island promoter methylation status in MCF7 and MDA-MB231 cells**. Shown are results of bisulfite genomic sequencing of 20 individual clones. The x axis of the graph indicates the CpG doublet position in the 5'-flanking region of *MsrB1 *with respect to the start site of transcription; the y axis indicates the percentage of the subclones that were methylated.

In order to evaluate epigenetic modifications of the *MsrB1 *promoter as a possible mechanism of *MsrB1 *silencing, MDA-MB231 cells were treated with 5-Aza-2'-deoxycytidine (5-Aza-dC). This compound generally showed reduced levels of DNA methylation and increased expression of genes silenced by DNA methylation [28]. The treatment of MDA-MB231 cell line lead to an increase in MsrB1 mRNA from basal levels as detected by RT-PCR (Figure 7). Interestingly, treatment with 5-Aza-dC of MCF7 cells did not have significant effects on the *MsrB1 *expression (data not shown). The effect of 5-Aza-dC on MsrB1 protein levels was then determined. An induction of MsrB1 protein by 5-Aza-dC was detected in low-expressing *MsrB1 *MDA-MB231 cells (Figure 7). Several studies suggested a synergistic effect of demethylation and histone deacetylase inhibition in re-expression of genes silenced by *de novo *methylation [29-31]. MDA-MB231 cells were treated with both 5-Aza-dC and trichostatin A (TSA), the last compound increased the levels of histone acetylation and expression of at least some target genes [32,33]. In our experimental conditions, TSA failed to reactivate *MsrB1 *in MDA-MB231 cells and did not potentiate the effect of 5-Aza-dC (data not shown). These findings suggested that DNA methylation of the *MsrB1 *promoter could be a mechanism responsible for the *MsrB1 *gene silencing observed in MDA-MB 231 cells. Sequencing analysis of bisulphite-modified DNA confirmed the increase of *MsrB1 *promoter methylation in MDA-MB231 compared to MCF-7 cells, *MsrB1 *gene promoter was indeed hypermethylated at the CG dinucleotide within the consensus Sp1-E binding site (Figure 8).

## Discussion

In the recent years, the interest in Msr system has focused on studies demonstrating the importance of this enzymatic system on the defence mechanism against oxidative damage. In particular, recent studies have reported the role of the Msr system in protecting lens and retinal cells against oxidative damage [34-36]. The existence of an association between increased levels of reactive oxygen species (ROS) and disturbed activities of enzymatic antioxidants in tumour cells [37] is well known; thus we began to study the Msr system in cancer cells.

Only a few reports were available on the transcriptional regulation of *MsrA/B *genes. Recently, we reported that human *MsrA *gene expression appeared to be different in several human cancer cell lines and gene promoter analysis showed a cell-specific *MsrA *transcriptional regulation [27]. In the present study, we reported evidence indicating a differential MsrB1 expression in two human cancer cell lines derived from the same tissue and we elucidated the mechanisms responsible for the *MsrB1 *gene expression. First we cloned the human *MsrB1 *5'-flanking promoter region and we provided evidence that this promoter fragment drives expression of luciferase-reporter constructs. The lack of TATA or CAAT boxes and a high content of CpG islands in the *MsrB1 *promoter are typical features of house-keeping genes. Interestingly, we found five Sp1 binding sites in the *MsrB1 *promoter and Sp1 was shown to be important for the transcription of genes with promoters that do not contain TATA boxes [38-40]. Indeed, we demonstrated the importance of Sp1 sites for *MsrB1 *promoter activity by site-directed mutagenesis. Three Sp1 binding sites located in the 169 base pair DNA fragment from the TSS showed the highest promoter activity. The progressive rise in the promoter activity seems to be directly correlated with the number of recognition sites for Sp1, suggesting its functional importance in *MsrB1 *gene expression. The longest fragment contains five Sp1 boxes but did not retain the highest activity suggesting the presence of possible *cis*-elements recognized by negative modulators.

In the present study, we showed evidence indicating a differential pattern of MsrB1 expression in breast cancer cell lines. Because of ubiquitous expression of Sp1, these sites, while involved in promoter activity, were not sufficient to explain the differences in endogenous content of the *MsrB1 *gene expression. Moreover, similar levels of Sp1 were detected in these cells by Western blot experiments.

Recently, it was reported that MsrA might have a role in *MsrB1 *transcription. In fact, a decrease of MsrB1 in the MsrA knockout mouse was observed [26]. However, we found comparable MsrA transcript and protein levels in both MDA-MB231 and MCF7 cells, suggesting that *MsrB1 *expression was regulated in a different way in our experimental system.

Overall, these findings suggested additional transcriptional mechanisms implicated in the *MsrB1 *silencing in MDA-MB231 cells.

Changes in DNA methylation patterns were identified in cancer and resulted in the silencing of important tumour suppressor genes involved in differentiation, apoptosis, cell cycle regulation, DNA repair and metastasis [41,42]. Moreover, several reports indicated that gene silencing was the consequence of DNA hypermethylation [43-45]. Recently, it was reported that several key genes are silenced in breast cancer and these events seemed to be linked to epigenetic modifications [46].

Consistent with an important role of DNA methylation in *MsrB1 *silencing, incubation of MDA-MB231 cells with 5-Aza-dC resulted in clearly detectable levels of both MsrB1 mRNA and protein. On the other hand, treatment of MDA-MB231 cells with TSA did not induce *MsrB1 *expression and there was no synergistic effect of TSA and 5-Aza-dC on *MsrB1 *expression. These findings suggested that transcriptional repression by DNA methylation was unlikely to depend upon a TSA-sensitive histone deacetylase. These observations are consistent with the recent reports studying the regulation of the hypermethylated genes *RFC*, *HPRT *and others. In these studies, the treatment of 5-Aza-dC induced the expression of these hypermethylated genes, whereas TSA treatment did not induce similar changes [47,48].

Previous studies reported that DNA methylation decreased Sp1 binding affinity to the respective promoter region [49,50]. In contrast, we show in this report, using ChIP experiments, that Sp1 binds the *MsrB1 *promoter region in both high-expressing MCF7 and low-expressing MDA-MB231 breast cancer cells. These findings suggest that Sp1 binding affinity to the *MsrB1 *promoter is not inhibited by DNA methylation. Similar data have been recently reported for the *LHR *promoter [51] and for the *CLDN4 *promoter [31].

A proposed mechanism to explain transcriptional inactivation from promoter methylation is based on the finding that methyl-CpG-binding proteins bind methylated DNA [52] and these proteins can then recruit a variety of transcriptional repressors [53-56]. Whether these transcriptional repressors are responsible to affect the *MsrB1 *expression in MDA-MB231 cells is still uncertain.

The epigenetic silencing of genes whose proteins function to attenuate oxidative free radicals, e. g., *GSTP1*, have been described in prostate cancer [57]. The epigenetic mediated loss of expression of antioxidant enzymes would create conditions favourable to both DNA base damage and accelerated proliferation. Obviously, more research is needed to postulate a possible *MsrB1 *involvement in metastatic process.

## Conclusion

In the present work, we showed that: (1) Sp1 is essential for the human *MsrB1 *promoter activity; (2) despite having all of the usual characteristics of a ubiquitously expressed "housekeeping" gene, *MsrB1 *is regulated differently among breast carcinoma cells; and (3) treatment with DNA methyltransferase inhibitor 5-Aza-dC induced *MsrB1 *gene expression in the highly metastatic MDA-MB231 cells. On the basis of these findings, we conclude that epigenetic changes are involved in *MsrB1 *silencing.

## Methods

### Cell cultures

Human breast cancer cells MDA-MB231 and MCF7 were grown in a humidified atmosphere containing 5% CO2 at 37°C in DMEM containing high glucose (4.5 g/liter at 25 mM) and supplemented with 50 units/ml penicillin, 50 mg/ml streptomycin, and 10% (vol/vol) FBS. For 5-Aza-2'-deoxycytidine (5-Aza-dC) and trichostatin A (TSA) treatment, cells were seeded at low density in a 100 mm tissue culture dish and maintained for a total 96 h. The former was added after 24 h in culture, and the cells were incubated with this drug for a total 72 h. Culture medium supplemented with 5-Aza-dC 10 μM was exchanged every 24 h. The latter was added to the media after 48 h of incubation and cells were then incubated with TSA for 24 h.

### Determination of 5'-terminal cDNA sequence

To map the transcription start site (TSS) of *MsrB1*, an RNA ligase-mediated rapid amplification of 5' end (RLM-RACE) strategy was used to obtain the full-length cDNA sequence at the 5' end, using a GeneRacerTM (Invitrogen) kit. A detailed protocol is available from the authors upon request. Briefly, five micrograms of total RNA from MCF7 cells were used to prepare 5'-racing cDNA as previously described [27]. RACE-1 primer (Table 1) was used in reverse transcription of adapter ligated mRNAs. The first-strand cDNA was amplified using the reverse gene-specific primer RACE-1 and the GeneRacerTM 5' Primer. An aliquot of PCR products was used as the template for a nested reaction using the RACE-2 reverse gene-specific primer and the GeneRacerTM 5' Nested Primer. PCR products were analyzed by 3% agarose, cloned into pCR4-TOPO vector and analyzed by sequencing.

### Construction of luciferase deletion plasmids

Genomic DNA was prepared from the MCF7 cells using Wizard Genomic DNA Purification Kit (Promega). Two oligonucleotides, P-1403 and RACE-1, were designed (Table 1), on the basis of genomic DNA sequence of 5'-flanking region of the MsrB1 gene, to amplify a portion of DNA starting -1276 bp upstream of the identified transcription start site (+1). In addition to the template (genomic DNA) and primers P-1403 and RACE-1, the reaction mixture contained 0.2 mM dNTPs, *Pfu *DNA polymerase buffer and 5 units of *Pfu *DNA polymerase (Promega), and was subjected to 35 cycles of amplification (60 s at 94°C, 60 s at 56°C, and 120 s at 72°C). The PCR product was recovered from low-melting agarose gel and used as template in PCR reaction. In this PCR amplification we used the primers P-1403-Hind and RACE-1-Hind tailed at 5' with a *Hind III *restriction endonuclease recognition sequence (Table 1). The PCR product was loaded on 1.5% low melting agarose gel, recovered from the gel, purified and ligated into pGL3-basic reporter vector *Hind III *digested and dephosphorylated; the resulting plasmid was designated P-1403. Insertion in the pGL3-basic reporter vector and the correct orientation were verified by DNA sequencing. A nested set of *MsrB1 *promoter fragments was created by PCR amplification using the oligonucleotide sequences shown in Table 1 (P-296, P-250, P-203 and P-165) and P-1403 as template. PCR amplification products were loaded on 2% low melting agarose gel, recovered from the gel, purified and ligated into pGL3-Basic reporter vector *Hind III *digested. The resulting plasmids, P-296, P-250, P-203 and P-165, were analyzed by DNA sequencing to ensure the fidelity of amplification and the correct orientation.

### Site-directed mutagenesis

The 296 bp (-169 bp to +127 bp) and the 203 bp (-76 bp to + 127 bp) promoter regions incorporated into the P-296 construct and into the P-203 construct respectively were subjected to site-directed mutagenesis, to eliminate cores of Sp1-C and SP1-E binding sites, using the Quick Change Site-Directed Mutagenesis Kit (Stratagene). The mutagenic primers (forSp1-C and revSp1-C) containing the mutation of Sp1-C were reported in Table 1. Both primers annealed to the same target sequence on opposite strands of P-296. Site-directed mutagenesis was performed according to the manufacturer and the resulting plasmid was designated P-296Sp1CMut. The two mutagenic primers containing the mutation of Sp1-E were also reported in Table 1. Both primers (forSp1-E and revSp1-E) annealed to the same target sequence on opposite strands of P-296, P296 Sp1CMut and P-203. The site-directed mutagenesis was performed as described by the manufacturer. The resulting plasmids were designated P-296 Sp1CMut, P-296 Sp1EMut, P-296Sp1C/E Mut and P-203 Sp1EMut and were confirmed by DNA sequencing.

### Transient transfections and luciferase assays

MDA-MB231 cells and MCF7 cells were plated in 12-well plates 24 h before transfection. The day of transfection, cells were washed twice with PBS solution and replaced with DMEM medium containing 1% FBS (not containing penicillin-streptomycin). Cells were transfected with 0.8 μg of P-1403 (the longest luciferase construct) and equimolar amounts for other plasmids were used. After 2 h of incubation at 37°C, the transfection solution was withdrawn and replaced with the complete medium described above, and cultivated for an additional period of 24 h at 37°C. Transfection were performed in duplicate, and repeated at least five times. Measurement of luciferase activity was performed 24 h after transfection using the Luciferase Assay Kit (Promega) according to the manufacturer's protocol. Each lysate was measured twice. Luciferase activities were normalized with respect to protein concentration in each extract to correct for transfection efficiency, and the reporter gene expression was expressed as relative light units. The luciferase activity of each construct was compared with that of the promoterless pGL3 Basic vector.

### RT-PCR

RT-PCR was performed using 1 μg of total RNA extracted from MDA-MB231 and MCF7 cell lines as described above. Reverse transcription reactions were performed using M-MLV Reverse Transcriptase (Sigma) and polydT primers, as recommended by the manufacturer. F1MsrB and R1MsrB primers (Table 1) were designed to amplify a portion of 200 bp of *MsrB1 *gene in PCR amplification reaction. In addition to primers and 2 μl of template (cDNAs obtained as reported above) the 50 μl reaction mixture contained 0.2 mM dNTPs, *Taq *DNA polymerase buffer and 5 units of *Taq *Gold DNA polymerase (Applied Biosystem, PE), and was subjected to 10 min at 95°C followed by 24 cycles of amplification (60 s at 95°C, 60 s at 54°C, and 60 s at 72°C). PCR products were verified by DNA sequencing. To provide further confidence in the data, 598 bp of glyceraldehyde-3-phosphate dehydrogenase (*GAPDH*) was amplified by 21 cycles of amplification using primers GAPDHf and GAPDHr (Table 1). *MsrA *amplification was obtained using MsrAfor 5'-GGAATGGGATGTTTCTGGG-3' and MsrArev 5'-TGAGCAGACTTCTTTATAAGTA-3' primers. The templates used in PCR reactions were the same cDNAs obtained as described above. The abundance of PCR products was measured by densitometric scanning of the ethidium bromide-stained agarose gels using Chemi Doc System, and the cDNA fragments corresponding to each amplified gene was compared between MDA-MB231 and MCF7 cells. Each data point was normalized using the quantity of GAPDH mRNA.

### Western blot analysis

Protein extracts were obtained from 80% confluent MDA-MB231 cells and MCF7 cells, using Passive Lysis Buffer (Promega). Crude extracts (40 μg) were analysed by SDS/PAGE on 15% (w/v) polyacrylamide gels. Proteins were transferred electrophoretically to nitrocellulose (for 120 min at 0.3 A) using an immunoblot transfer apparatus (Bio-Rad). After transfer, the nitrocellulose was incubated for 180 min at room temperature in 10% (w/v) non fat milk in Tris-buffered saline (TBS; 500 mM NaCl and 20 mM Tris/HCl, pH 7.5), supplemented with 0.05% (v/v) Tween 20, to block non-specific binding. The blot was incubated overnight at 4°C with 10% non fat milk in TBS, supplemented with 0.05% (v/v) Tween 20, containing mouse monoclonal anti-MsrB (Abcam Inc.) at a dilution of 1:1000. After three washes with TBS containing 0.05% (v/v) Tween 20, the blot was incubated for 60 min at room temperature with peroxidase-conjugated goat anti-mouse immunoglobulin (Calbiochem) diluted at 1:2000 in 10% non fat milk in TBS, supplemented with 0.05% (v/v) Tween 20. The blot was again washed three times with TBS containing 0.05% (v/v) Tween 20. Antibodies were visualized using a chemiluminescence detection system (Western Blotting Luminol Reagent, Santa Cruz Biotechnology). Western blotting analysis was also performed using rabbit polyclonal antiserum anti-MsrA produced in our laboratory as previously described [27] and rabbit polyclonal anti-Sp1 (Santa Cruz, sc-14027).

### Chromatin immunoprecipitation assay

To study protein/DNA interactions we used ChIP-IT™ Kit (Active Motif). A detailed protocol is available from the authors upon request. Briefly, MCF7 and MDA-MB231 cells were crosslinked for 10 min at room temperature by adding Fixation solution. Cells were scraped in presence of Cell Scraping Solution. Nuclei release was obtained with dounce homogenizer. The cells were centrifuged at 5000 rpm for 10 min at 4°C to pellet the nuclei. The nuclei pellet was resuspended in Shearing Buffer, supplemented with protease inhibitors and sonicated using ten pulses of 20 s. After centrifugation, the supernatant was diluted in ChIP buffer and pre-cleared with Protein G beads for 2 hours at 4°C. An aliquot (10 μl) of pre-cleared chromatin was stored at -20°C as "Input DNA". The supernatant was divided into three aliquots. No antibodies were added to one aliquot (negative control), and either RNA pol II antibody or Sp1-specific antibody (Santa Cruz, sc-14027 X) was added to the others two aliquots and they incubated overnight at 4°C on a rotating wheel. Protein G beads were added into each of the antibody/chromatin incubations and the tubes were incubated on a rotator for 1.5 hours at 4°C. Immunoprecipitated DNA was eluted from the washed Protein G beads and cross-linking was reversed by heating the elutes at 65°C overnight. The elutes were then digested with proteinase K at 42°C for 2 hours. DNA was purified using mini-columns provided with the ChIP-IT™ Kit. The MsrB1 promoter region was amplified by PCR using 5-GGCCCAGGAGTGGTCC-3' (forward) and 5'-CCAACTGACCAAAGGCTGC-3'(reverse) primers.

### DNA methylation analysis of the *MsrB1 *gene

Genomic DNA (0.5 μg in a volume of 100 μl) was denaturated in 0.2 M NaOH at 37°C for 10 min and incubated with 3 M sodium bisulfite at 55°C for 16 h. After bisulfite treatment, DNA was desalted using the Wizard DNA Clean-up System (Promega), ethanol-precipitated, washed, and resuspended in TE (100 μl). Bisulfite-modified DNA was amplified by PCR with a primer set designed to detect both methylated and unmethylated promoter regions of the *MsrB1 *gene. Primer sequences for the unmethylated reaction were 5'-GGCCCAGGAGTGGTCC-3' (forward) and 5'-GAAGCTGCAGAACGACATGG-3' (reverse), and for the methylated reaction were 5'- GGTTTAGGAGTGGTTTTAAGGAG-3' (forward) and 5'-AAAACTACAAAACGACATAA-3' (reverse). Primers were located at -123 (forward) and +127 (reverse) from the transcription start site. Purified PCR products were cloned into the TOPO-TA vector (Invitrogen) by using the manufacturer's standard protocol. Twenty clones were sequenced from each sample.

## Abbreviations

RFC: reduced folate carrier

HPRT: hypoxanthine-guanine phosphoribosyl transferase

LHR: luteinizing hormone receptor

CLDN4: claudin-4

GSTP1: pi-class glutathione S-transferase

## Authors' contributions

ADL and BF conceived, designed and coordinated the study, and wrote the manuscript. ADL and MN created the various reporter constructs and conducted the transient transfection experiments. All authors participate in analysis of the results. CDI and PS participated in the coordination and helped write the manuscript.

## Figures and Tables

**Table 1:** Sequences of all the primers used in this study

RACE-1	5'-GAAGCTGCAGAACGACATGG-3'
RACE-1-Hind	5'-CCCAAGCTTGAAGCTGCAGAACGACATGG-3'
RACE-2	5'-CCAACTGACCAAAGGCTGC-3'
P-1403	5'-GAGAGTCTTGATCTTGTCGC-3'
P-1403-Hind	5'-CCCAAGCTTGAGAGTCTTGATCTTGTCG-3'
P-296	5'-CCCAAGCTTGGAGATGCGATTGGACTGG-3'
P-250	5'-CCCAAGCTTGGCCCAGGAGTGGTCC-3'
P-203	5'-CCCAAGCTTAAGATCGTATTGGACCAGCC-3'
P-165	5'-CCCAAGCTTTCCCGGAGGCGGAAGCC-3'
F1MsrB	5'-ATGTCGTTCTGCAGCTTCTTC-3'
R1MsrB	5'-CACACTTGCCACAGGACAC-3'
GAPDHf	5'-CCACCCATGGCAAATTCCATGGCA-3'
GAPDHr	5'-TCTAGACGGCAGGTCAGGTCCACC-3'
forSp1-C	5'-GGACTGGCACCGGGGGTTTTGGCCTTGGGGAAGTGG-3'
revSp1-C	5'-CCACTTCCCCAAGGCCAAAACCCCCGGTGCCAGTCC-3'
forSp1-E	5'-CCAGCCCTGAGGAGGGTTTTGGCCTCCCGGAGGCGG-3'
revSp1-E	5'-CCGCCTCCGGGAGGCCAAAACCCTCCTCAGGGCTGG-3'
